# Differential patterns of human parvovirus B19 seropositivity among diabetic and febrile children and adolescents: evidence from the Aseer region of Saudi Arabia

**DOI:** 10.3389/fped.2026.1706955

**Published:** 2026-03-16

**Authors:** Ghanem Ali Al-Shahrani, Ahmed Mossa Al-Hakami, Yahya Mohamed Shabi, Abdulah Jarboa Alqahtani, Abdelwahid Saeed Ali

**Affiliations:** 1 Department of Microbiology and Serology, King Abdullah Hospital, Bisha, Saudi Arabia; 2 Department of Microbiology and Clinical Parasitology, College of Medicine, King Khalid University, Abha, Saudi Arabia

**Keywords:** age, B19V, children, fever, gender, seropositivity, T1DM

## Abstract

**Introduction:**

Human parvovirus B19 (B19V) is a significant causative agent of a diverse range of clinical manifestations in children. It is the primary cause of pediatric “erythema infectiosum,” also known as “fifth disease.” The virus can also cause hydrops fetalis during in-utero infections. Global serosurveys indicate that approximately 50% of individuals acquire protective immunity against the virus by the age 15; however, seroprevalence data vary depending on the geographic region and population studied.

**Methodology:**

In the present study, we report epidemiological data on B19V infectivity among pediatric patients in the southern region (Aseer region) of the Kingdom of Saudi Arabia (KSA). A cross-sectional survey was conducted among 437 pediatric patients (225 males and 212 females) attending major regional hospitals in the Aseer region of Saudi Arabia. Serum samples were tested for B19V-specific IgG and IgM antibodies using an indirect ELISA system. The seroprevalence of the virus was evaluated, and its association with age, gender, and clinical conditions (including diabetes and febrile illness) among children was analyzed.

**Results:**

The overall seropositivity rates for B19V-specific IgG and IgM were 32.5% and 6.2%, respectively, while 2.8% of the total tested patients were positive for both IgG and IgM antibodies. Anti-B19V IgG seropositivity increased significantly with age among the children (*p* = 0.001), whereas no significant differences in anti-B19V IgM seropositivity were observed across the different age groups (*p* = 0.193). Furthermore, anti-B19V IgG seropositivity was significantly higher among patients with type 1 diabetes mellitus (T1DM) compared with those with other clinical conditions. The IgG seroprevalence rates were 40.1%, 18.8%, and 38.3% among T1DM, febrile, and other pediatric patients, respectively. In contrast, no statistically significant association was observed between anti-B19V IgM seropositivity and underlying health condition, with corresponding rates of 3.1%, 9.4%, and 8.3% among diabetic, febrile, and other pediatric patients, respectively.

**Discussion:**

These findings suggest cumulative childhood exposure to the virus, even though no major outbreaks were reported in the region during the study period. The results also underscore the need for larger, community-based surveillance studies to enable timely outbreak detection and the implementation of appropriate control measures to limit virus transmission.

## Introduction

Human parvovirus B19 (B19V) is a small, non-enveloped, single-stranded DNA virus belonging to the genus *Erythroparvovirus,* subfamily *Parvovirinae,* within the family *Parvoviridae* (1). The virus was first recognized in 1974 by Cossart et al. while evaluating assays for hepatitis B virus surface antigen (2). The virus is infectious across all age groups of people, including neonates, infants, children, adults, and the elderly (3). Children infected with B19V typically present with “erythema infectiosum,” a mild, self-limiting condition. This illness is also known as “fifth disease,” as it shares clinical similarities with the four well-known classic childhood exanthems: measles, scarlet fever, rubella, and Dukes’ disease. B19V exhibits strong tropism for erythroid progenitor cells in the bone marrow, leading to transient suppression of erythropoiesis and, in certain populations, the development of severe anemia (4, 5). Transmission of B19V occurs primarily via respiratory droplets, blood or blood product transfusion, and maternal–fetal vertical transmission (6–8). While B19V infection is usually mild in people who are otherwise healthy, complications can occur among people with underlying blood disorders (9) or weakened immune systems (10). Following infection, anti-B19V IgM antibodies peak and provide definite evidence of recent infection, becoming detectable within 7–10 days after exposure. In contrast, the presence of anti-B19V IgG antibodies alone indicates past exposure to the virus and confers long-term immunity (11). Despite extensive global data available on B19V infection, seroepidemiological profiles remain incomplete in many regions across the world, and the influence of age and clinical status on seropositivity varies based on region (12, 13). Some previous investigations have examined the relationship between B19V infection and type 1 diabetes mellitus (T1DM) in children, but the findings have been inconclusive. O'Bryan et al. first raised the possibility of a link by describing the virus infection in children with new-onset T1DM (14). However, other serological studies have indicated that no concrete association exists between B19V infection and the development of T1DM (15, 16).

In Saudi Arabia, some serological surveys have been conducted to determine the prevalence of B19V infection in the eastern (17), western (18, 19), and central (20) provinces of the country, contributing substantially to the understanding of the epidemiology of the infection in these regions. However, the epidemiological profiles and clinical phenotypes of B19V infections in the southern provinces of Saudi Arabia, particularly the Aseer region—the largest and most densely populated area of the country—have not been previously investigated. Based on these considerations, the present study was designed as a cross-sectional seroprevalence survey of B19V infection among pediatric patients. In addition, the associations between B9V-specific IgG and IgM seropositivity and patient characteristics, such gender, age, and clinical conditions, were analyzed. To the best of our knowledge, this is the first investigation to assess the seroprevalence of B19V in this region, particularly among pediatric patients. Moreover, it represents the first regional dataset intended to inform public health surveillance and support clinical-decision making.

## Methods

### Study design and population

A cross-sectional study was conducted among children attending major hospitals in the Aseer region of Saudi Arabia between June 2021 and February 2022. A total of 437 children (aged ≤18 years) were enrolled from multiple healthcare facilities, including Aseer Central Hospital (ACH), Abha Maternity and Childhood Hospital (AMCH), Khamis Mushait General Hospital (KGH), Regional Diabetes Center (RDC), and Mahayil General Hospital (MGH). Eligible participants included children presenting for follow-up of diabetes mellitus, evaluation of febrile illness, or other medical reasons. Exclusion criteria included recent vaccination, diagnosed immune disorders that could alter antibody responses, and refusal of parental or guardian consent. Ethical approval was obtained from the Research Ethics Committee at King Khalid University (HAPO-06-B-001; Approval No. ECM#2020-3310, dated 4 January 2021). Written informed consent was obtained from parents or guardians before participation.

### Samples collection

Venous blood samples (3–5 mL) were collected in plain tubes under aseptic conditions from all enrolled participants. The samples were allowed to clot at room temperature and were then centrifuged at 3,000 rpm for 10 min to separate the sera. Serum aliquots were transferred into cryogenic vials (Merck, Darmstadt, Germany) and stored at −70 °C until testing.

### Detection of anti-B19V IgM and IgG antibodies

Serological testing for B19V-specific IgM and IgG antibodies was performed using commercially available indirect enzyme-linked immunosorbent assay (ELISA) kits (Virion/SERION, Würzburg, Germany), in accordance with the manufacturer's instructions. Serum samples were diluted 1:100 and analyzed alongside positive, negative, and blank controls. Absorbance was measured bichromatically at 405 nm, with a reference wavelength of 620–690 nm, using an ELISA reader (Humareader, Human, Wiesbaden, Germany).

### Interpretation of results

Results for B19V-specific IgG and IgM were registered and interpreted based on optical density (OD) values obtained in relation to the test kit manufacturer-defined cut-off values. Samples with OD readings above the cut-off were considered positive, indicating the presence of B19V-specific antibodies, whereas samples with OD values below the cut-off were interpreted as negative, reflecting the absence of detectable antibodies. IgG positivity was interpreted as evidence of previous exposure to B19V or, in infants under 1 year of age, residual maternally derived antibodies. IgM positivity was interpreted as suggestive of recent active infection. Samples with OD values falling at or near the cut-off were considered equivocal and regarded as inconclusive. They were excluded from the seroprevalence calculations and comparative statistical analyses.

### Quality control

Each assay run included internal quality controls. Serum samples positive for IgM and/or IgG were additionally screened for rheumatoid factor using a commercial latex agglutination test (Human, Wiesbaden, Germany) to exclude the false-positive reactions.

### Statistical analysis

Data were analyzed using IBM SPSS Statistics, version 26 (IBM Corp., Armonk, NY, USA). Descriptive statistics were presented as frequencies and percentages for categorical variables, and as mean, median, and range for continuous variables. Associations between demographic or clinical groups and B19V IgG/IgM serostatus were examined using Pearson's chi-square test or Fisher's exact test when expected cell counts were <5. A two-tailed *p*-value of <0.05 was considered statistically significant. Graphical presentations, including distribution plots and comparative visualizations, were generated using Python, version 3.11.8. Despite comparisons between the various subgroups, adjustments for multiple comparisons were not applied in this study.

## Results

### Baseline characteristics of the study population

A total of 437 children were included in the analysis, of whom 225 (51.5%) were boys and 212 (48.5%) were girls. The age distribution was as follows: 67 (15.3%) were ≤1 year old, 119 (27.2%) were 2–6 years, 167 (38.2%) were 7–12 years, and 84 (19.2%) were 13–18 years. With respect to clinical groups, 174 (39.8%) were diabetic patients, 106 (24.3%) were febrile patients, and 157 (35.9%) were categorized as other patients (Table 1).

**Table 1:** Baseline demographic and clinical characteristics of the enrolled children.

Variable	Category	No. of children, *n* (%)
Gender	Male	225 (51.5)
	Female	212 (48.5)
Age group	≤1 year	67 (15.3)
	2–6 years	119 (27.2)
	7–12 years	167 (38.2)
	13–18 years	84 (19.2)
Clinical conditions	T1DM patients	174 (39.8)
	Febrile patients	106 (24.3)
	Other patients	157 (35.9)

Total no. 437.

### Serological findings

Based on the serological interpretation, 267 (61.1%) children were B19V-specific IgG negative, 28 (6.4%) equivocal and 142 (32.5%) were positive.

For B19V-specific IgM, a total of 381 (87.2%) children were negative, 29 (6.6%) equivocal, and 27 (6.2%) positive (Table 2).

**Table 2:** Descriptive analysis of B19V IgG and IgM optical density (OD) values and serological results (*N* = 437).

Variable	Mean ± SD	Median (range)	Negative, *n* (%)	Equivocal, *n* (%)	Positive, *n*o (%)
B19V-IgG	0.36 ± 0.49	0.111 (0.000–1.969)	267 (61.1)	28 (6.4)	142 (32.5)
B9V-IgM	0.20 ± 0.22	0.148 (0.010–2.393)	381 (87.2)	29 (6.6)	27 (6.2)

### Gender and B19V seropositivity

Analysis of serological outcomes by gender revealed no statistically significant differences in the prevalence of B19V IgG or IgM antibodies between male and female patients. The proportion of IgG seropositivity was comparable across genders, with no evidence of association (*p* > 0.05).

Similarly, IgM seropositivity did not differ significantly by gender (*p* > 0.05). When examining combined IgG/IgM positivity, the distribution remained balanced between boys and girls, and no statistically meaningful difference was observed.

### Association between B19V seropositivity and age in children

When stratified by age group, IgG seropositivity varied significantly across different age categories of patients (*p* = 0.001). It was detected in 19 of 61 (31.1%) children aged ≤1 year, 26 of 113 (23.0%) aged 2–6 years, 56 of 154 (36.4%) aged 7–12 years, and 41 of 81 (50.6%) aged 13–18 years.

**Figure 1:**
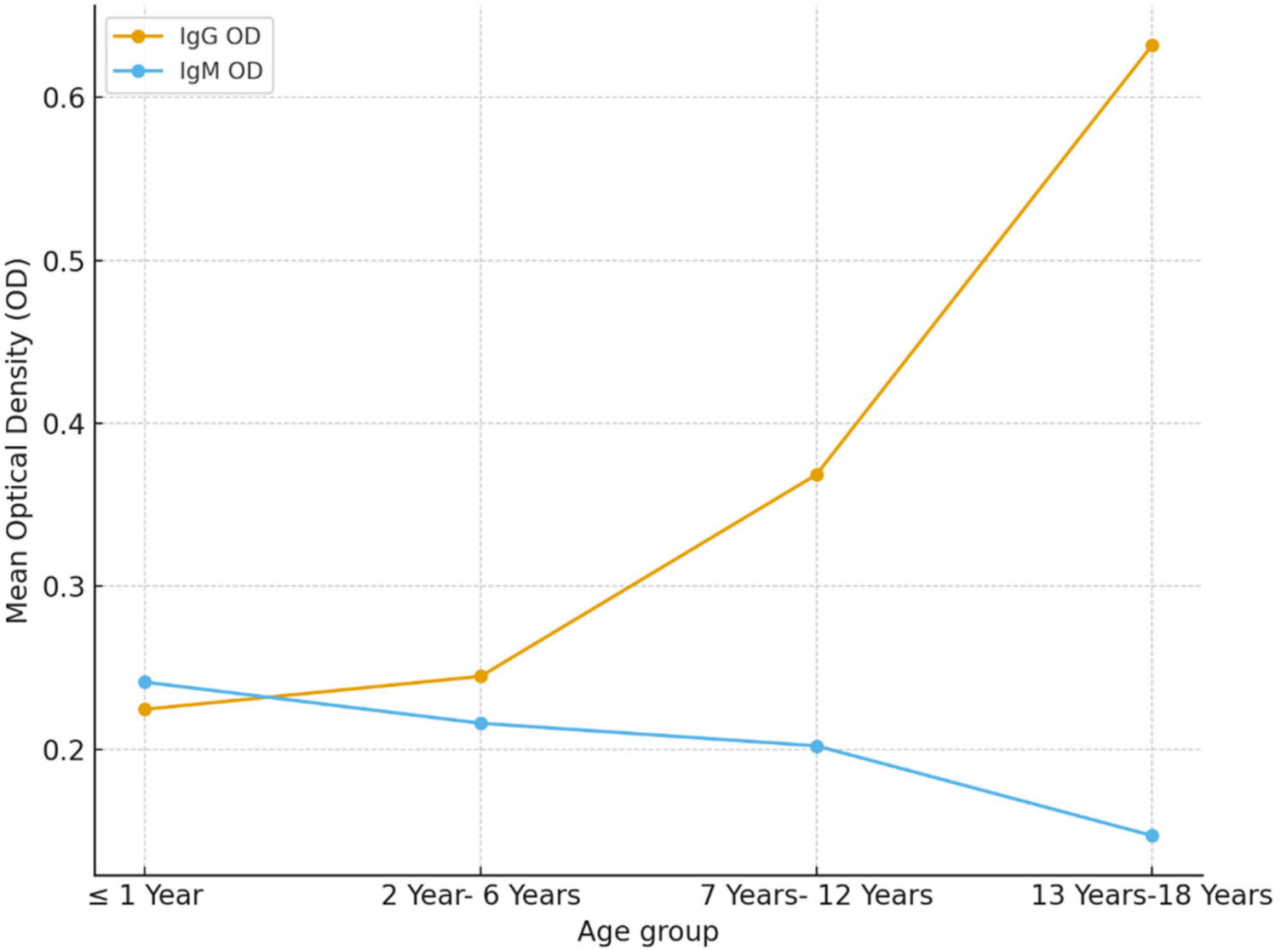
Mean optical density (OD) values of B19V IgG and IgM across pediatric age groups in the Aseer region (*N* = 437).

In contrast, IgM seropositivity did not differ significantly among age groups (*p* = 0.193). It was detected in 5 of 63 (7.9%) children aged ≤1 year, 9 of 106 (8.5%) aged 2–6 years, 12 of 159 (7.5%) aged 7–12 years, and 1 of 80 (1.2%) aged 13–18 years (Table 3; Figure 1).

**Table 3:** Association between age groups and B19V IgG and IgM seropositivity among children in the Aseer region of Saudi Arabia.

B19V-Ig	Age group	Negative, *n* (%)	Positive, *n* (%)	Total, *n* (%)	*p*-Value
B19V-specific IgG	≤1 year	42 (68.9)	19 (31.1)	61 (14.9)	0.001*
	2–6 years	87 (77.0)	26 (23.0)	113 (27.6)	
	7–12 years	98 (63.6)	56 (36.4)	154 (37.7)	
	13–18 years	40 (49.4)	41 (50.6)	81 (19.8)	
	Total	267 (65.3)	142 (34.7)	409 (100)	
B19V-specific IgM	≤1 year	58 (92.1)	5 (7.9)	63 (15.4)	0.193
	2–6 years	97 (91.5)	9 (8.5)	106 (26.0)	
	7–12 years	147 (92.5)	12 (7.5)	159 (39.0)	
	13–18 years	79 (98.8)	1 (1.3)	80 (19.6)	
	Total	381 (93.4)	27 (6.6)	408 (100)	

B19V, human parvovirus B19; Ig, immunoglobulin; IgG, immunoglobulin G; IgM, immunoglobulin M.

* *p*-Value < 0.05 is considered statistically significant.

### Association between B19V seropositivity and clinical conditions in children

Among the various clinical groups, IgG seropositivity was highest among diabetic patients, with 67 of 164 (40.1%) testing positive, compared to 18 of 96 (18.8%) febrile patients and 57 of 149 (38.3%) classified as other patients. The differences were statistically significant (*p* = 0.001).

For IgM, 5 out of 164 (3.1%) diabetic patients, 9 of 96 (9.4%) febrile patients, and 13 of 148 (8.3%) other patients tested positive. No significant differences in IgM seropositivity were observed across the three groups of patients (*p* = 0.148) (Table 4).

**Table 4:** Association between clinical conditions and B19V IgG and IgM seropositivity among children in Aseer region.

B19V-immunoglobulin	Clinical group	Negative, *n* (%)	Positive, *n* (%)	Total, *n* (%)	*p*-Value
B19V-specific IgG	Diabetic patients	97 (59.1)	67 (40.9)	164 (40.1)	0.001*
	Febrile patients	78 (81.3)	18 (18.8)	96 (23.5)	
	Other patients	92 (61.7)	57 (38.3)	149 (36.4)	
	Total	267 (65.3)	142 (34.7)	409 (100.0)	
B19V-specific IgM	Diabetic patients	159 (97.0)	5 (3.1)	164 (39.8)	0.148
	Febrile patients	87 (90.6)	9 (9.4)	96 (24.3)	
	Other patients	135 (91.2)	13 (8.8)	148 (35.9)	
	Total	381 (93.4)	27 (6.6)	408 (100.0)	

B19V, human parvovirus B19; Ig, immunoglobulin; IgG, immunoglobulin G; IgM, immunoglobulin M.

* *p*-Value < 0.05 is considered statistically significant.

## Discussion

Human parvovirus B19 (B19V) is an infectious agent responsible for erythema infectiosum in children, particularly among those with blood disorders, and may result in complications such as hydrops fetalis upon vertical transmission during pregnancy (3–5, 6). The present study was conducted to assess the seroprevalence rates of B19V among pediatric patients in the Aseer region (south of Saudi Arabia), representing the first report from this study area. The overall seroprevalence rates of B19V-specific IgG and IgM reported in this study were 32.5% and 6.2%, respectively. The IgG prevalence rates are considered relatively high as compared with several previous studies (19, 20) but low with respect to other investigations (6, 21, 22), whereas IgM prevalence rates are almost comparable with previously reported rates. These findings indicate the spread of the virus in southern Saudi Arabia, despite the absence of major outbreaks during the study period, and highlight the need for larger community-based surveillance and help to impose control measures to stop the spread of the virus. The variations in the B19V seroprevalence across studies can be explained by the differences in the sensitivity of the detection methods employed, the sample size of the patients tested, and the underlying health conditions targeted in each study. The relatively low prevalence rate of anti-B19V-IgM detected in this study (6.2%) is similar to those detected previously, which can generally be attributed to the short-lived nature of immunoglobulin during virus infection (23). However, this does not imply a limited spread of the virus in the study region or the country at large.

Our study also revealed that B19V seropositivity varies among the different age groups of children. Infants (≤1 year old) showed high prevalence rates of IgG seropositivity (31.1%). This finding does not indicate that infants were infected with the virus; rather, it most likely reflects transplacental transfer of maternal IgG antibodies. The presence of these antibodies may suggest a relatively high incidence of infection among pregnant women in the study population (24, 25). Again, this underscores the need for routine testing for virus infectivity as part of gynecological care. Moreover, it seems necessary to conduct a nationwide survey on virus infection in pregnant women. Notably, B19V IgG responses detected among children under 1 year of age may indicate residual maternal immunity and do not necessarily represent an active virus infection in this group of the children. While this is generally true for IgG responses, it is unlikely to be the case for IgM responses. IgG seropositivity was noted to increase with the age of children, and almost half (50.6%) of the adolescents tested were B19V IgG positive. These findings complied with most previously published reports (19, 26). Despite its detection in all age groups, our study demonstrated no significant variations in B19V IgM seropositivity among the different age groups of pediatric patients (*p* = 0.193). This allowed us to infer that an acute infection may have occurred among the tested children despite no obvious clinical symptoms being reported during or before sampling. It was confirmed that detection of B19V IgM was associated with a history of blood transfusion, pregnancy, and neoplastic conditions (27–30). Our findings also concurred with those al-Frayh and co-workers (21), who concluded that Saudi people have been exposed to B19V early in life and that the incidence of exposure increases with age. In the present study, no significant statistical differences were observed in the prevalence of B19V IgG or IgM antibodies between male and female patients. This conforms with previously published reports indicating that B19V infectivity and serological responses do not have ethnic, socioeconomic, gender, or geographic boundaries (4). However, clinical manifestations induced may vary by gender. It had been confirmed in many studies that B19V causes more arthropathy cases among adult female compared with male patients (31, 32).

A novel observation from this study is that children with diabetes mellitus type 1 (T1DM) exhibited the highest B19V IgG seroprevalence (40.1%), whereas children presenting with febrile illnesses demonstrated slightly the highest IgM positivity (9.4%). This suggests that children with chronic diseases such as diabetes may have accumulated greater lifetime exposure to B19V, while febrile children likely reflect acute or recent infections. While earlier studies have emphasized the risk of B19V-related complications in patients with hemolytic, immunological (4, 6, 10, 20, 33), and neoplastic disorders (22, 34), reports specifically examining seroprevalence in diabetic children are scarce. Our findings may therefore represent a novel, hypothesis-generating association, warranting further investigation in larger cohort studies, particularly among pediatric diabetic patients. In this study, anti-B19V IgM antibodies were detected in patients with all clinical manifestations, particularly those with febrile conditions. Fever is a cardinal sign during the acute phase of virus infectivity. These findings align with emerging evidence that B19V infection is not confined to classical presentations, but is also associated with manifestations including viral fevers (35), anemias (11, 17), meningoencephalitis (19, 36), and Guillain–Barré syndrome (37, 38), predominantly in immunocompromised hosts. This underscores the expanding clinical spectrum of B19V and highlights the need for heightened clinical awareness, particularly among vulnerable populations. From a public health perspective, the detection of both past and recent B19V infections in children emphasizes the importance of considering the virus in the differential diagnosis of febrile illnesses, anemia, and rash in pediatric practice. Moreover, evidence of increasing B19V outbreaks globally highlights the need for continued serological and molecular surveillance of B19V infections in Saudi Arabia, particularly given the paucity of local data. Pregnant women, patients with hematological or immunological disorders, and children with T1DM remain high-risk populations in whom B19V screening may have clinical value.

### Strengths and limitations

A key strength of this study is its multicenter design, encompassing children of diverse ages and clinical backgrounds, thereby enhancing the representativeness of the findings. However, the study is limited by its cross-sectional design, which precludes assessment of temporal or causal relationship. Reliance on serological testing without molecular confirmation may underestimate recent B19V infections, due to the short detection window of IgM antibodies. Equivocal results were excluded from inferential analyses in accordance with standard practice; however, if the proportion of equivocal results differed across subgroups, small biases may persist. The observed age-related increase in IgG seropositivity likely reflects cumulative exposure to B19V, although interpretation in infants under 1 year is limited by the presence of transplacentally acquired maternal IgG. The observed enrichment of IgG among diabetic children and IgM among febrile children is hypothesis-generating, with differences in care pathways and healthcare contact potentially contributing to these contrasts. In addition, multiple subgroup comparisons were conducted without formal adjustment for multiplicity, and findings should therefore be interpreted as exploratory. Finally, our sampling period (June 2021 to February 2022) predates the strongest global resurgence signals reported in 2023–2025; therefore, such comparisons are intended as epidemiologic context rather than evidence of causal attribution.

## Conclusions

This study documented a considerable level of B19V seropositivity among children in the Aseer region (southern Saudi Arabia). The seropositive findings were observed across different groups with respect to gender, age, and underlying health conditions. Although an association between B19V IgG responses and diagnosed T1DM cases was observed, this should not be interpreted as evidence of disease causation. Nevertheless, it represents a novel observation that has not previously been reported in the region. These results help address an important local evidence gap and underscore the need for ongoing surveillance and heightened clinical awareness, in line with reports from the Gulf region and recent global outbreaks.

## Data Availability

The original contributions presented in the study are included in the article/Supplementary Material, further inquiries can be directed to the corresponding author.
